# Ground-based adaptive horsemanship lessons for veterans with post-traumatic stress disorder: a randomized controlled pilot study

**DOI:** 10.3389/fpsyt.2024.1390212

**Published:** 2024-05-28

**Authors:** Ellen M. Rankins, Andrea Quinn, Kenneth H. McKeever, Karyn Malinowski

**Affiliations:** ^1^ Equine Science Center, Department of Animal Sciences, Rutgers University, New Brunswick, NJ, United States; ^2^ Center for Psychological Services, Rutgers University, New Brunswick, NJ, United States

**Keywords:** PTSD, trauma, equine-assisted services, horse, veteran, cortisol, nervous system, oxytocin

## Abstract

**Introduction:**

Equine-assisted services (EAS) has received attention as a potential treatment strategy for post-traumatic stress disorder (PTSD), as existing literature indicates that symptoms may decrease following EAS. Relatively little is known about the mechanisms at play during lessons and if physiological measures are impacted. The objectives of this pilot study were to 1) explore the effects of adaptive horsemanship (AH) lessons on symptoms of PTSD, hormone concentrations, and social motor synchrony; 2) determine if physiological changes occur as veterans interact with horses; and 3) explore if the interaction between veteran and horse changes over the 8-week session.

**Methods:**

Veterans with PTSD were randomly assigned to control (CON, n = 3) or AH (n = 6) groups for an 8-week period (clinical trial; NCT04850573; clinicaltrials.gov). Veterans completed the PTSD Checklist (PCL-5) and Brief Symptom Inventory (BSI) at pre-, post-, and 2- and 6-month follow-up time points. They also completed a social motor synchrony test (pendulum swinging) and blood draw at pre- and post-time points. In weeks 1, 4, and 8, blood samples were drawn at 0 min, 3 min, 5 min, 25 min, and 30 min during the 30-min AH lessons. Veterans completed the Human–Animal Interaction Scale (HAIS) after each lesson. Blood samples were assayed for plasma cortisol, epinephrine, norepinephrine, and oxytocin. Data were analyzed with repeated measure ANOVAs. Changes in PTSD symptoms from pre- to post-time point were analyzed with paired t-tests.

**Results:**

Changes in PCL-5 scores tended to differ (*p* = 0.0989), and global BSI scores differed (*p* = 0.0266) between AH (−11.5 ± 5.5, mean ± SE; −0.5 ± 0.2) and CON (5.3 ± 5.4; 0.4 ± 0.2) groups. Social motor synchrony and hormone concentrations did not differ between groups or time points (*p* > 0.05). Cortisol, norepinephrine, and oxytocin concentrations did not differ across sessions (*p* > 0.05). Epinephrine concentrations tended (*p* = 0.0744) to decrease from week 1 to 4 of sessions. HAIS scores increased (*p* ≥ 0.0437) in week 3 and remained elevated as compared to week 1.

**Discussion:**

Participant recruitment was the greatest challenge. These preliminary results agree with the literature suggesting that EAS can reduce symptoms of PTSD.

## 1 Introduction

Post-traumatic stress disorder (PTSD) is characterized by changes in behavior and functioning that fall within four symptom clusters—re-experiencing, avoidance, cognition and mood, and arousal (1). These symptoms develop after exposure to a traumatic event such as threatened death, serious injury, or sexual violation (1). The US Department of Veterans Affairs (2) estimates that 11%–30% of US veterans experience PTSD (3). Current treatment options for PTSD include medication and therapies such as cognitive processing therapy (CPT) and eye movement, desensitization, and restructuring (EMDR). The current standard for treatment is considered therapy, and in recent years, medication prescription has declined (4). Dropout rates among veterans in therapy are high (5), which has led to an exploration of alternative and complementary treatment options for PTSD. Among these are equine-assisted services.

Equine-assisted services (EAS) is an umbrella term encompassing therapies incorporating horses, their movement, or their environment; adaptive horsemanship; and equine-assisted learning, which share the common goal of improving people’s health and well-being through interactions with horses (6). Therapies that incorporate horses can include occupational therapy, physical therapy, speech language pathology, counseling, psychotherapy, and recreational therapy (6). In all these instances, the therapy must be provided by a licensed therapist who incorporates horses into their professional practice. Adaptive horsemanship is provided by a riding instructor or other equine professional, generally credentialled by a certifying organization. This category includes adaptive equestrian sport, adaptive or therapeutic riding, and ground-based horsemanship instruction (6). The common goal of these adaptive horsemanship activities is to further the participant’s riding and horsemanship skills. Equine-assisted learning is the newest area of EAS and, thus, is slightly less well-defined than the other two areas (6). Programming in this area focuses on school-related skills and individual and organizational development.

A recent review on equine-assisted services for veterans with a history of trauma reported that the existing literature indicates veterans can benefit from this type of programming (7). The author also concluded that the field is in the early stages of scientific development with more studies needed. Studies that have investigated the impacts of equine-assisted services on veterans with PTSD have focused on psychotherapy or counseling incorporating horses and adaptive horsemanship, generally adaptive riding. Among veterans, PTSD symptoms as measured on self-report and clinician administered scales and related measures such as depression, quality of life, anxiety, and affect are reported to improve following psychotherapy or counseling incorporating horses (8–19) and adaptive horsemanship (20–26).

There have also been preliminary investigations into the effects of equine-assisted services on veterans’ salivary cortisol, functional MRI (fMRI), heart rate, heart rate variability, respiration rate, and blood pressure (8, 9, 14, 20). These types of physiological and neural measures are of interest as they can be useful biomarkers and begin to elucidate potential mechanisms of change. Most of these studies have focused on taking measures at pre- and post-intervention time points leaving a lack of knowledge concerning the physiological responses occurring during lessons as veterans interact with the horses.

This type of knowledge can help further the development of theoretical models to explain mechanisms of change during equine-assisted services. Currently, there are several proposed theories and ideas for why equine-assisted services can be beneficial to individuals who have experienced trauma. These include self-determination theory, the horse–human relationship and the bonding that can occur between the two, enhanced engagement and therapeutic alliance during sessions, emotional and physiological mirroring, self-distancing through metaphor, and an opportunity for biophilia and mindfulness (7, 27).

The autonomic nervous system controls most of the body’s autonomic functions such as blood circulation and digestion. There is a dynamic balance between the sympathetic and parasympathetic divisions of the autonomic nervous system. Increased sympathetic activation is associated with greater arousal (increased heart rate, norepinephrine, and epinephrine and decreased heart rate variability), whereas parasympathetic activation results in lowered arousal. The autonomic nervous system can be modulated by factors outside of arousal. Oxytocin has been implicated as one of these modulators and is released in response to positive social contact and bonding (28–30). The other endocrine system commonly used as a marker of stress is the hypothalamic–pituitary–adrenal (HPA) axis. Cortisol is the end product of this system and is elevated under conditions of acute and chronic stress.

Muscular tension, most often in the area of the jaw, neck, and shoulder, has also been proposed as a potential indicator of stress in humans (31–33). Its applicability in an applied setting has not been studied, and it may prove a useful, non-invasive tool for measuring stress when recordings are made using surface electromyography (sEMG).

Altered patterns of social functioning are a product of PTSD and can have major deleterious effects on an individual’s homelife and integration into society (34). PTSD is often also co-morbid with major depressive disorder with similar events leading to an increased risk for both disorders (35). Co-regulation is altered in those with depression (36). Social motor synchrony is a readily measurable form of co-regulation in which the synchrony between gross motor movements is measured (36–38). Social motor synchrony is a potential marker of an individual’s health and their ability to experience attunement or synchronization with those around them and thus could prove to be a relevant biomarker for those with PTSD (39).

Documenting and investigating the interactions between horse and human during equine-assisted services is needed, as the horse and its interactions with the human are key to these services. Therapist–client alliance and human–horse bond have been shown to increase over 12 weeks of therapy but is unknown how soon this change occurs and if it remains consistent (11). Self-reports of interactions between human and animal, such as the Human–Animal Interaction Scale are one means of beginning to explore how the interactions between veterans and horses may change over the course of a session (40).

Based on the gaps in our current understanding of the effects of equine-assisted services on veterans with PTSD, four objectives for this pilot study were developed:

1) Determine the feasibility of recruiting, enrolling, and completing data collection with veterans with PTSD.
2) Explore the effects of adaptive horsemanship lessons on symptoms of PTSD, hormone concentrations, and social motor synchrony in combat veterans.
3) Determine if physiological changes occur as veterans interact with horses in weekly lessons.
4) Explore if the interaction between veteran and horse changes over the course of an 8-week session.

## 2 Materials and methods

Study procedures and materials were approved by the Rutgers University Institutional Review Board (Protocol No. 2019001999) and Institutional Animal Care and Use Committee (Protocol No. 999900214). Study procedures took place in Piscataway, NJ (pre-, post-, and follow-up measures) and Monroe Township, New Jersey (adaptive horsemanship lessons). The study was considered a clinical trial and registered (NCT04850573) at clinicaltrials.gov.

### 2.1 Study design

A randomized controlled design was used in which participants were randomly assigned to an 8-week wait-listed control (CON) or 8-week adaptive horsemanship (AH) condition.

### 2.2 Study conditions

#### 2.2.1 Wait-listed control

The CON group continued their daily activities with no changes for the 8-week period and were offered 4 hours of adaptive horsemanship lessons after completing their post measures.

#### 2.2.2 Adaptive horsemanship lessons

Veterans in the AH group participated in 30-min lessons once a week for 8 weeks with the same horse or pony (n=6). Horses and ponies had been selected and trained for work in equine-assisted services following Professional Association of Therapeutic Horsemanship, International (PATH Intl) guidelines. Horses and ponies continued to work in their regularly scheduled programming (adaptive riding, physical and occupational therapy integrating horses, and equine-assisted learning) outside of study activities. Horse behavior and physiology were monitored throughout the study, and no adverse effects were noted (see Rankins et al. (41) for a full description).

All lessons were taught and overseen by one individual who is certified by PATH Intl as an Equine Specialist in Mental Health and Learning (ESHML). In the first lesson, veterans were instructed on basic horse safety considerations (range of vision, approaching, and working around the horse), horse behavior (flight or fight response and prey animals), and grooming (standard grooming procedure including cleaning the hooves). Instruction was provided verbally, and then, the participant practiced skills such as approaching the horse and grooming with feedback as needed from the instructor.

Subsequent lessons began with the veteran grooming the horse. The horse was secured with a halter and lead line to a tie ring during all grooming. After grooming, the participant received direct instruction on a horsemanship skill (leading, leading with the horse at liberty in the arena, or long lining) or reviewed the previously introduced skill. The remainder of the lesson was allocated to allowing the participant to practice the skill with feedback from the instructor as needed to keep the horse and participant safe and allow them to progress. Within each skill, progression proceeded from performing the skill on straight lines and circles to obstacle courses with simple obstacles (ground poles, cones, and barrels). Not all veteran and horse pairs progressed through all skills, as progress was tailored to the individual.

### 2.3 Participants

Nine (30% of targeted enrollment of 30) male veterans between the ages of 18 and 75 who had been deployed and experienced combat completed data collection (**Table 1**). Potential participants with severe traumatic brain injury, an amputation, a diagnosis or experience of bi-polar disorder, schizophrenia, or substance dependence in the last 3 months or a pacemaker were excluded (**Table 2**).

**Table 1:** Demographics of participants who completed all data collection procedures.

	Veteran Participants		Non-veteran Participants (n = 9)
	CON (n = 3)	AH (n = 6)	
Age	35 ± 6	38 ± 3	35 ± 3.5
Race			
White	3	5	7
Black/African American	0	1	1
Asian	0	0	1
Hispanic or Latino			
Yes	0	1	0
No	3	5	9
Education Level			
Associate’s Degree	0	1	1
Some College	2	1	3
Bachelor’s Degree	1	1	2
Master’s Degree	0	3	3
Years of Military Service	12 ± 7	12 ± 4	Not applicable
Service Branch			
Army	1	4	Not applicable
Marine Corps	2	2	Not applicable
History of Traumatic Brain Injury			
No	0	6	Not applicable
Yes (mild or moderate)	3	0	Not applicable
Previous Experience with Horses			
No	2	4	Not applicable
Yes	1	2	Not applicable
Number of Medications	3.7 ± 3.7	2.5 ± 0.8	0.4 ± 0.4
Number of PTSD Treatments	1.3 ± 0.9	1.2 ± 0.3	Not applicable

Continuous data are presented as means ± SE. Categorical data are presented as counts.

**Table 2:** Inclusion and exclusion criteria for veteran and non-veteran participants.

Criteria for Veterans		Criteria for Non-veterans	
Inclusion	Exclusion	Inclusion	Exclusion
• Male • Veteran of the United States Military • Deployment in a military combat zone and combat experience • 18–75 years of age	• Severe traumatic brain injury (TBI) • An amputation • A diagnosis or experience of bi-polar disorder, schizophrenia, or substance dependence in the previous 3 months. • A pacemaker	• Male • 18–75 years of age	• Service in the United States Military • A chronic mental or physical health issue • An acute mental or physical health issue in the previous 3 months • A pacemaker

An additional nine male non-veterans between the ages of 18 and 75 were enrolled to serve as the other half of the dyad in the social motor synchrony task in which pairs of participants swing a pendulum and the synchrony between pendulums is recorded (see *Section 2.5.1* for more details). Synchrony can only be measured in dyads, and the standard for this type of task is to use a healthy individual as the other part of the dyad (37, 38). Non-veterans were excluded if they had a chronic or acute mental or physical health issue or a pacemaker (**Table 2**). Dyads were pair-matched on the basis of age (non-veteran within ± 5 years of the veteran’s age) and highest level of education completed following the methods of Varlet et al. (38).

### 2.4 Recruitment and screening

**Figure 1:**
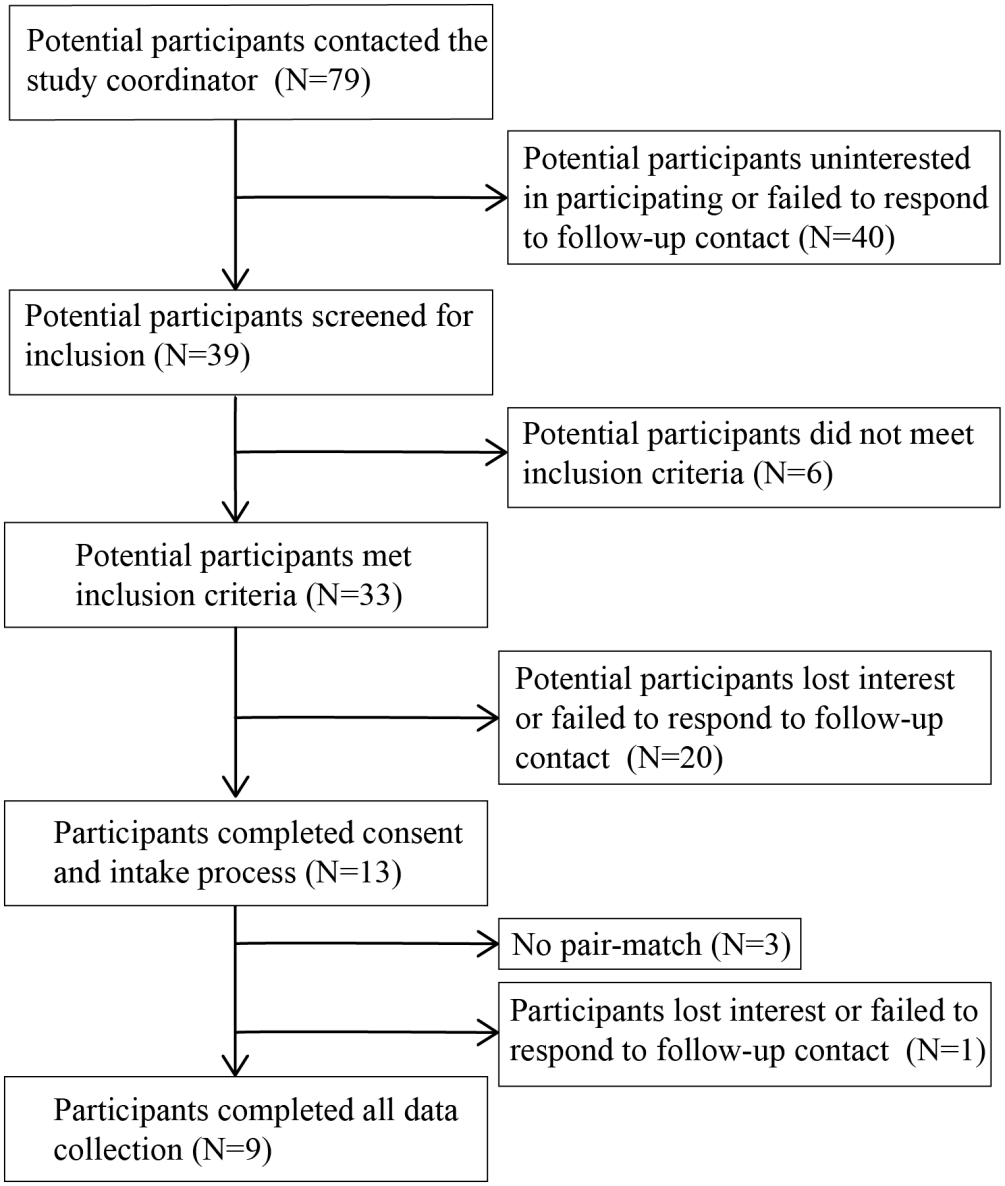
Flow diagram of number of veteran participants and their participation in study activities.

**Figure 2:**
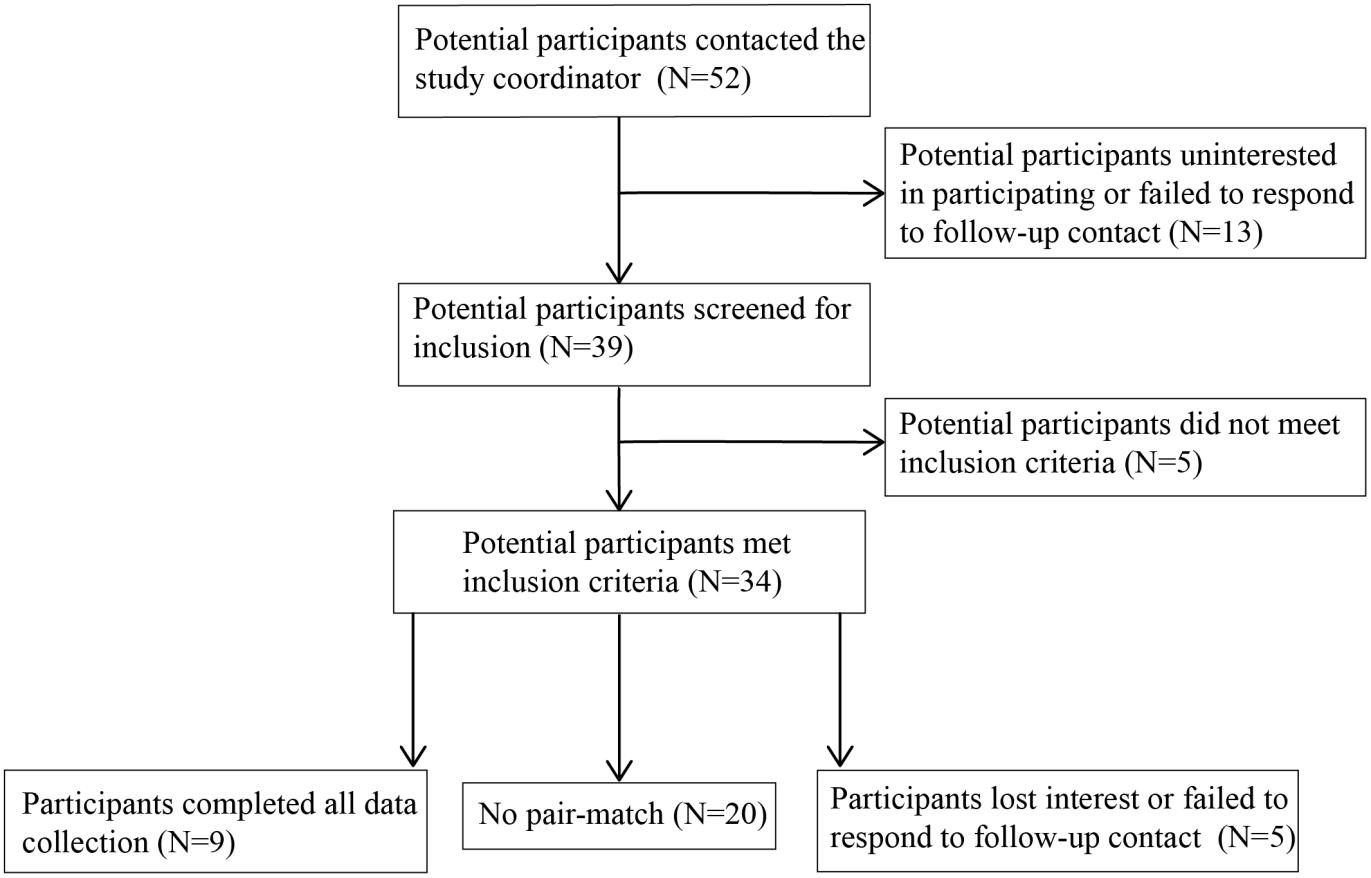
Flow diagram of number of non-veteran participants and their participation in study activities.

Potential participants were recruited from the surrounding area through word of mouth, distribution of flyers through the New Jersey Department of Military and Veteran Affairs, and radio advertisements. Those interested in participating (n = 79 veterans, n = 52 non-veterans; **Figures 1**, **2**) contacted the study coordinator (EMR) to learn more and complete a pre-screening questionnaire over the phone to assess eligibility criteria (**Table 1**). Non-veterans meeting the eligibility criteria were placed on a waiting list until a veteran with whom they could be matched enrolled in the study.

If veterans met the eligibility criteria in the pre-screening questionnaire, they completed the informed consent process with the study coordinator and the Life Events Checklist (LEC-5) and Clinician Administered PTSD Scale (CAPS-5) interview with a trained graduate student from the Rutgers University School of Applied and Professional Psychology (42–45). Students were overseen by a clinical psychologist. If students were unsure how to score an item given the information provided by the veteran, the issue was discussed with the clinical psychologist and a score agreed upon. Veterans meeting clinical cutoffs for PTSD on the past month version of the CAPS-5 (n= 13; **Figure 1**) were enrolled in the study.

### 2.5 Pre-, post-, and follow-up measures

Pre-measures were taken the week before veterans started the 8-week adaptive horsemanship (AH) program or CON period. Post-measures were taken the week following completion of the AH program or CON period. Follow-up measures were taken 2 and 6 months after completion of the AH program. At the pre-measure time point, non-veteran participants arrived 20 minutes before veteran participants to complete the informed consent process and provide demographic information. Only veteran participants completed the follow-up measures. Pre-measure procedures consisted of a social motor synchrony test, blood draw, and completion of the PTSD Checklist (PCL-5) and brief symptom inventory (BSI). These procedures were repeated at the post-measure time point, and veteran participants also completed a questionnaire on medication, treatment, and lifestyle changes. At the follow-up time points, only the PCL-5, BSI, and questionnaire on medication, treatment, and lifestyle changes were completed.

#### 2.5.1 Social motor synchrony

Participants were seated in chairs (0.9 m apart) in a quiet room to complete the social motor synchrony test adapted from Fitzpatrick et al. (37). Each chair had a pendulum (54 cm length, 100 g weight) attached to it with a telemetric triaxial gyroscope (Ultium EMG, Noraxon, Scottsdale, AZ, USA) secured (3M, St. Paul, MN, USA) to the end of the pendulum. Data were recorded continuously and transmitted to a computer (Mobile Precision 3541, Dell^®^, Round Rock, TX, USA) for real-time monitoring. Markers denoting the start and end of each data collection phase were placed automatically by the software (myoMUSCLE™, Noraxon, Scottsdale, AZ, USA). Participants completed two tests: unintentional and intentional social motor synchrony. Unintentional social motor synchrony was assessed in a 90-s test where participants swung their pendulums at a self-selected, comfortable pace. Participants looked away from one another for the first 30 s, at one another for the middle 30 s, and away from one another for the final 30 s. This was repeated three times for three weight configurations: 1) veteran’s weight in the middle and non-veteran at the bottom, 2) both weights at the bottom, and 3) veteran’s weight at the bottom and non-veteran weight in the middle. Intentional social motor synchrony was assessed in a test consisting of 60 s of data collection followed by 30 s of rest and washout. In the rest and washout periods, participants could sit and rest. Participants swung their pendulums in an antiphase (pendulums at opposite points in the swinging arcs) and in-phase (pendulums at the same point in the swinging arcs) trials. Trials were repeated three times within each of the three pendulum weight configurations. Trial order was randomized across participants.

The first and last 10 s of each trial were removed before data analysis. The continuous relative phase time series of the data was calculated using the Hilbert transformation (37, 38, 46). The circular variance of continuous relative phase time series was calculated (MATLAB^®^ 2022b; MathWorks, Natick, MA, USA) to determine synchrony between participants (47).

#### 2.5.2 Blood draw and hormone assays

A registered nurse performed blood draws via antecubital fossa venipuncture (Vacutainer Safety-Lok blood collection set, 23 GA, Becton, Dickinson and Company, Franklin Lakes, NJ, USA) after scrubbing the site with a 70% isopropyl alcohol solution and allowing it to air dry. Blood was drawn into lithium heparin-coated (2 mL) and EDTA-coated tubes (3 mL) and immediately placed on ice until centrifuged at 2,500 rpm for 5 min. Plasma was aliquoted into cryovials and stored at −80°C until assayed.

Plasma samples were thawed at room temperature (20°C–22°C) and assayed for cortisol (heparinized), oxytocin (EDTA), norepinephrine (EDTA), and epinephrine (EDTA) concentrations. Cortisol concentrations were determined using a competitive enzyme-linked immunosorbent assay (Cortisol ELISA, Enzo Life Sciences Inc., Farmingdale, NY, USA) validated for use in humans. A 1:16 dilution was used for all samples. The intraassay coefficient of variation was 2.9%, and the interassay coefficient of variation across three runs was 6.1%. Analytical sensitivity was 56.72 pg/mL. Epinephrine concentrations were determined using a competitive enzyme-linked immunosorbent assay (LDN^®^, Nordhorn, Germany) validated for use in humans. Plasma sample volumes were between 180 µL and 200 µL. The intraassay coefficient of variation was 4.4%, and the interassay coefficient of variation across four runs was 11.4%. Analytical sensitivity was 0.05 ng/mL. Norepinephrine concentrations were determined using a competitive enzyme-linked immunosorbent assay (LDN^®^, Nordhorn, Germany) validated for use in humans. Plasma sample volumes were between 180 µL and 200 µL. The intraassay coefficient of variation was 3.7%, and the interassay coefficient of variation across four runs was 10.6%. Analytical sensitivity was 0.02 ng/mL. Oxytocin concentrations were determined using a competitive enzyme-linked immunosorbent assay (Oxytocin ELISA, Enzo Life Sciences Inc., Farmingdale, NY, USA) validated for use in humans. A 1:8 dilution was used for all samples. The intraassay coefficient of variation was 2.3%, and the interassay coefficient of variation across three runs was 4.2%. Analytical sensitivity was 15.0 pg/mL. All hormone concentrations were calculated using an immunoassay software package (MARS Data Analysis Software v 3.20, BMG Labtech, Cary, New York) from a four-parameter logistic regression. Curve fit was high across all assays and runs (R^2^ ≥ 0.99).

#### 2.5.3 PTSD symptoms

Veterans completed the PCL-5 and BSI, self-report measures to monitor the severity of current (past week) PTSD symptoms and psychological distress (48–51). A trained graduate student from the Rutgers University School of Applied and Professional Psychology scored all questionnaires.

### 2.6 Measures during adaptive horsemanship lessons

Data were also collected during the adaptive horsemanship lessons from the veterans enrolled in the adaptive horsemanship condition (n=6). Blood samples were drawn in weeks 1, 4, and 8. Surface electromyography (sEMG) and responses on the Human–Animal Interaction Scale were collected in weeks 1–8.

#### 2.6.1 Blood samples and hormone assays

In weeks 1, 4, and 8, a registered nurse inserted an intravenous catheter (forearm or antecubital fossa, Insyte™ Autoguard™, 22 GA, 25 mm, Becton, Dickinson and Company, Franklin Lakes, NJ, USA) after scrubbing the site with a 70% isopropyl alcohol solution and allowing it to air dry. Catheters were placed at least 20 min prior to the start of the lesson. Blood samples were drawn into lithium heparin- (2 mL) and EDTA-coated tubes (3 mL) at 0 min (start of the lesson), 3 min, 5 min, 25 min, and 30 min (end of the lesson) into the lesson. Lines were flushed with a saline solution (0.9% NaCl) following each blood draw. Catheters were capped and covered with gauze in between blood draws to keep them clean. Samples were immediately placed on ice until centrifuged at 2,500 rpm for 5 min. Plasma was aliquoted into cryovials and stored at −80°C until assayed.

Plasma samples were thawed at room temperature (20°C–22°C) and assayed for cortisol (heparinized), oxytocin (EDTA), norepinephrine (EDTA), and epinephrine (EDTA) concentrations as described in *Section 2.5.2*.

#### 2.6.2 Surface electromyography

Electrode attachment sites (right and left *masseter, sternocleidomastoid*, and upper *cervical trapezius*) were scrubbed with a 70% isopropyl alcohol solution and allowed to air dry. Electrodes (Ag/AgCl, 1.3 cm diameter, 2 cm inter-electrode distance, Noraxon, Scottsdale, AZ, USA) were placed parallel to the muscle fibers along the muscle belly approximately 2.5 cm above the mandibular angle (*masseter*), 3 cm below the earlobe (*sternocleidomastoid*), and along the line from the lateral edge of the acromion to the seventh cervical vertebrae (C7) (upper *cervical trapezius*) (52–54). Electrodes were attached to telemetric transmitter units (Ulitum, EMG, Noraxon, Scottsdale, AZ, USA), which were secured with self-adhesive backing (Noraxon, Scottsdale, AZ, USA).

Telemetric units connected wirelessly to a laptop (Mobile Precision 3541, Dell^®^, Round Rock, TX, USA) where data were viewed in real time and collected continuously (sampling frequency of 2,000 Hz, analog gain of 500, MR 3.14™, Noraxon, Scottsdale, AZ, USA). The data collected is a trace of electrical activity within the muscle of interest that reflects activation of the muscle fibers. Data collection only proceeded if impedance as measured by the software system was below 10 kΩ. As data were collected, a high-pass band filter of 10 Hz and a lowpass band filter of 500 Hz were applied to remove noise.

In post-processing, signals were filtered using a Butterworth filter (20 Hz cutoff) and rectified (MyoMUSCLE™, Norazon, Scottsdale, AZ, USA) (55). Peak amplitude over a 100-ms window during voluntary contraction in muscles of interest was used for normalization. Participants were instructed to clench their jaw, hold a shoulder shrug, and flex their neck to elicit muscular contraction. Signals were normalized to the peak value obtained in this period. Frequency content of the sEMG signal was determined using a Fast Fourier transformation (FFT) (MyoMuscle™, Noraxon, Scottsdale AZ, USA). The average rectified value (ARV, %) and median frequency (MF, Hz) were calculated (MyoMuscle™, Noraxon, Scottsdale AZ, USA) for 100-ms periods at 2.5 min, 7.5 min, 12.5 min, 17.5 min, 22.5 min, and 27.5 min into the lesson as a measure of muscle activity during those time periods.

#### 2.6.3 Human–Animal Interaction Scale

Veterans completed the Human–Animal Interaction Scale [(40) Qualtrics, Seattle, WA, USA] at the end of each lesson on a tablet (iPad mini 2, OS v12.5.4, Apple, Cupertino, CA). The 24-item scale was designed to describe and quantify human and animal behaviors during interaction with one another. The scale has high reliability and re-test reliability with acceptable validity (40). Responses were exported to a spreadsheet (Excel™, Microsoft, Redmond, WA, USA). The sum of undesirable human behaviors (two items) was subtracted from the sum of desirable human behaviors (12 items) to obtain the human subscore. The sum of undesirable animal behaviors (four items) was subtracted from the sum of desirable animal behaviors (six items) to obtain the animal subscore. The human and animal subscores were summed to obtain the total score.

### 2.7 Statistical analysis

Data were inspected for normality, and those violating the assumptions of normality were analyzed using an alternative distribution in the statistical model. The most appropriate models were selected using inspection of the residual plots and comparison of Akaike Information Criterion (AIC) values. Statistically different means in ANOVAs were separated using Tukey’s method. Significance was set at *p* < 0.05.

Circular variance values from the test of social motor synchrony and hormone concentrations from pre- and post-measure time points were analyzed with a repeated measures mixed model ANOVA with fixed effects of time point, treatment, and their interaction and a random effect of participant (SAS 9.4, Cary, NC, USA). Time point was considered a repeated measure.

Changes in scores and subscores from the PCL-5 and BSI from pre- to post-measure time point were analyzed using an independent sample t-test with equal variance (SAS 9.4, Cary, NC, USA). Scores from the follow-ups (EAA group only) were analyzed with a repeated measures mixed model ANOVA with a fixed effect of time point and a random effect of participant.

ARV and MF of the sEMG signals were analyzed with a mixed model, repeated measures, lognormal distribution ANOVA with fixed effects of week, time point, side, and their interactions and a random effect of participant. Plasma hormone concentrations during the lessons were analyzed using mixed model, repeated measures ANOVAs with fixed effects of week, time point, and their interactions and a random effect of participant. Week and time point were considered repeated measures. Subscores and total scores from the Human–Animal Interaction Scale were analyzed using a mixed model, repeated measures ANOVA with a fixed effect of week and a random effect of participant.

## 3 Results

Nine veterans completed data collection with 100% attendance at pre- and post-measure data collections and adaptive horsemanship lessons. Three completed the wait-list control, and six completed the ground-based adaptive horsemanship lessons.

### 3.1 PTSD symptoms

The change in total scores from the PCL-5 and scores from the cluster of cognition and mood alteration symptoms (Cluster D) tended to differ between AH and CON groups with decreases in the AH group and increases in the CON group (*p* = 0.0989 and 0.0889) (**Table 3**). The change in global severity scores and somatization and psychoticism subscores from the BSI differed significantly between groups with scores decreasing in the AH group and increasing in the CON group (*p* ≤ 0.0488) (**Table 3**). The change in depression, hostility, and paranoid ideation subscores from the BSI tended to differ between groups with scores decreasing in the AH group and increasing in the CON group (*p* ≤ 0.0721).

**Table 3:** Changes in PTSD symptoms from pre- to post-measure time points as measured on the PTSD Checklist (PCL-5) and Brief Symptom Inventory (BSI).

	Δ in Score from Pre- to Post-Measure		*p*-value
	CON (n = 3)	AH (n = 6)	
PCL-5	5.3 ± 5.4	−11.5 ± 5.5	*0.0989*
Reexperiencing	1 ± 1.7	−2.2 ± 1.4	0.2131
Avoidance	0.3 ± 1.7	−1.2 ± 0.4	0.2654
Cognition and Mood Alterations	3 ± 2.3	−5.5 ± 2.8	*0.0889*
Hyperarousal	1 ± 2.5	−4.3 ± 1.7	0.1203
Global BSI	0.4 ± 0.2	−0.5 ± 0.2	**0.0266**
Somatization	3 ± 0.6	−1.3 ± 1.2	**0.0488**
Obsessive Compulsive	2.7 ± 1.2	−2.8 ± 2.6	0.1801
Interpersonal Sensitivity	1 ± 1.5	−3.2 ± 1.8	0.1822
Depression	4.7 ± 3.3	−2.5 ± 1.6	*0.0579*
Anxiety	2 ± 1.7	−1.5 ± 1.6	0.2306
Hostility	3.3 ± 1.8	−3.8 ± 2.1	*0.0616*
Phobic Anxiety	0 ± 0	7.2 ± 7.5	0.5792
Paranoid Ideation	1.7 ± 0.7	−2.3 ± 1.3	*0.0721*
Psychoticism	2 ± 1.5	−4.7 ± 1.3	**0.0165**

Data are presented as means ± SEs. Bold font indicates a statistically significant difference between groups. Italic font indicates a trend toward statistically significant differences between groups.

Obsessive–compulsive, anxiety, and paranoid ideation subscores from the BSI were significantly lower (*p* = 0.0414, 0.0271, and 0.0456) in veterans in the AH group at 2 months post-intervention as compared to the pre-time point (**Table 4**).

**Table 4:** PTSD symptom severity as measured on the PTSD Checklist (PCL-5) and Brief Symptom Inventory (BSI) at pre-, post-, and follow-up measure time points in veterans in the adaptive horsemanship (AH) group.

	Pre	Post	2-Month Follow-up	6-Month Follow-up
PCL-5	51.8 ± 4.9	40.3 ± 8.7	43.3 ± 7.5	40.3 ± 6.9
Reexperiencing	11.2 ± 1.4	9.0 ± 2.1	9.3 ± 1.6	8.2 ± 1.4
Avoidance	5.5 ± 0.7	4.3 ± 0.7	4.8 ± 0.9	4.5 ± 1.2
Cognition and Mood Alterations	17.5 ± 3.1	12.0 ± 3.4	13.8 ± 3.3	13.7 ± 3.0
Hyperarousal	17.7 ± 1.8	13.3 ± 2.8	15.3 ± 2.5	14.0 ± 2.3
Global BSI	1.9 ± 0.3	1.5 ± 0.3	1.6 ± 0.4	1.7 ± 0.4
Somatization	8.8 ± 2.3	7.5 ± 2.9	6.5 ± 3.5	9.8 ± 3.9
Obsessive Compulsive	18.8 ± 2.0 ^a^	16.7 ± 3.3 a^,b^	12.2 ± 3.4 ^b^	13.3 ± 3.2^a,b^
Interpersonal Sensitivity	7.8 ± 1.6	4.7 ± 1.3	4.0 ± 1.4	5.3 ± 2.2
Depression	12.7 ± 2.8	10.2 ± 3.4	9.3 ± 2.8	9.8 ± 3.5
Anxiety	11.0 ± 2.4^a^	9.5 ± 2.6^a,b^	6.7 ± 2.2^b^	8.2 ± 2.3^a,b^
Hostility	10.7 ± 2.0	6.8 ± 2.1	6.3 ± 1.9	9.5 ± 2.6
Phobic Anxiety	10.7 ± 1.5	8.8 ± 1.6	7.8 ± 2.2	8.7 ± 2.6
Paranoid Ideation	9.5 ± 1.3^a^	7.2 ± 1.8^a,b^	4.3 ± 1.5^b^	6.5 ± 2.0^a,b^
Psychoticism	10.7 ± 2.5^a^	6.0 ± 1.6^b^	6.5 ± 1.7^a,b^	6.5 ± 1.9^a,b^

Data are presented as means ± SEs. ^a,b^Differing superscripts within a row indicate statistically significant differences (*p* < 0.05).

### 3.2 Social motor synchrony

**Figure 3:**
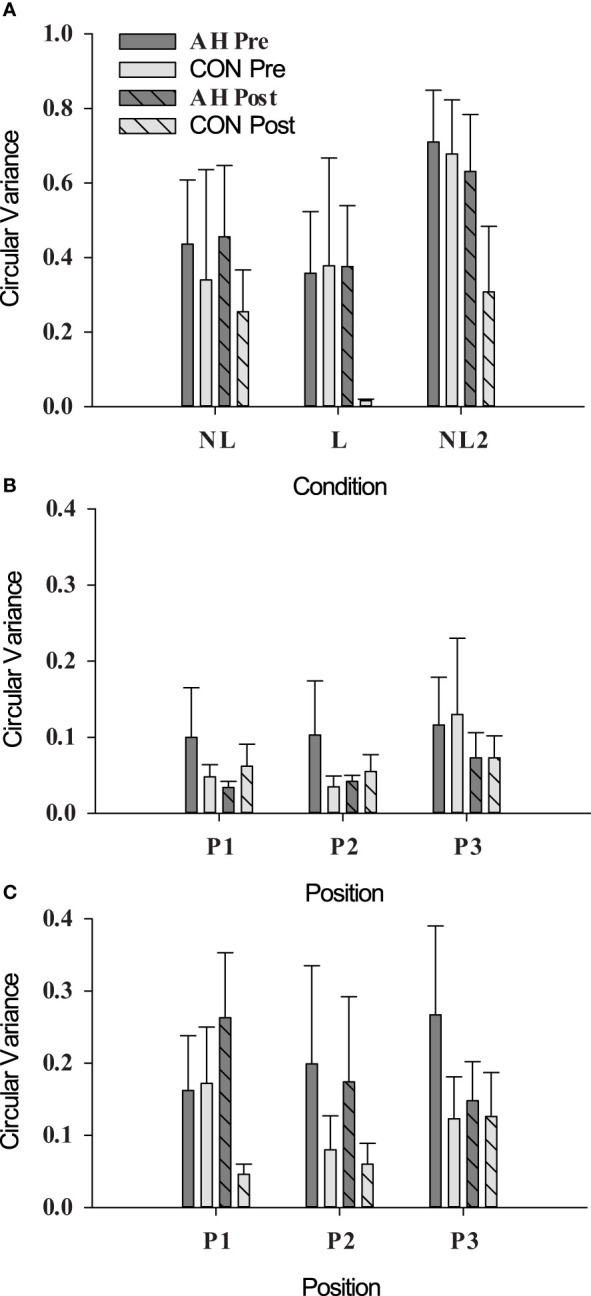
Circular variance as measured during social motor synchrony tests under **(A)** unintentional (NL, not looking; L, looking), **(B)** intentional in-phase (position refers to pendulum position, see *Section 2.5.1*), and **(C)** intentional antiphase conditions in AH and CON groups at pre- and post-measure time points. Data are presented as means ± SEs.

There were no significant differences (*p* ≥ 0.2151) in circular variance across time points or groups (**Figure 3**).

### 3.3 Plasma hormone concentrations

There were no significant differences (*p* ≥ 0.1432) in cortisol, epinephrine, norepinephrine, or oxytocin concentrations across pre- and post-measure time points or groups (**Table 5**).

**Table 5:** Plasma hormone concentrations in veterans enrolled in the AH and CON groups at pre- and post-measure time points.

	AH (n = 6)		CON (n = 3)	
	Pre	Post	Pre	Post
Cortsiol (µg/dL)	3.79 ± 1.26	1.95 ± 0.47	1.54 ± 0.44	1.23 ± 0.44
Epinephrine (ng/mL)	0.08 ± 0.03	0.05 ± 0.01	0.04 ± 0.01	0.05 ± 0.01
Norepinephrine (ng/mL)	0.43 ± 0.06	0.44 ± 0.06	0.29 ± 0.02	0.43 ± 0.10
Oxytocin (µg/mL)	0.07 ± 0.01	0.06 ± 0.01	0.04 ± 0.01	0.04 ± 0.00

Data are presented as means ± SEs.

During AH lessons, there were no significant differences (*p* ≥ 0.2748) in cortisol, norepinephrine, or oxytocin concentrations across time points within lessons or weeks (**Table 6**). Epinephrine concentrations trended toward a statistically significant difference (*p* = 0.0744) in weeks 1 and 4 with concentrations being lower in week 4 (**Table 6**).

**Table 6:** Plasma hormone concentrations in veterans during AH lessons.

	Time Point	Cortsiol (µg/dL)	Epinephrine (ng/mL)	Norepinephrine (ng/mL)	Oxytocin (µg/mL)
Week 1					
	0 min	6.57 ± 2.42	0.11 ± 0.03	0.63 ± 0.16	0.07 ± 0.01
	3 min	5.02 ± 0.92	0.07 ± 0.02	0.59 ± 0.14	0.07 ± 0.01
	5 min	5.22 ± 0.87	0.08 ± 0.02	0.66 ± 0.12	0.08 ± 0.02
	25 min	4.70 ± 1.18	0.09 ± 0.03	0.64 ± 0.14	0.07 ± 0.01
	30 min	3.75 ± 0.77	0.11 ± 0.03	0.56 ± 0.08	0.08 ± 0.02
Week 4					
	0 min	5.72 ± 2.32	0.05 ± 0.01	0.83 ± 0.10	0.07 ± 0.02
	3 min	7.53 ± 2.83	0.05 ± 0.01	0.94 ± 0.12	0.06 ± 0.01
	5 min	3.63 ± 0.56	0.05 ± 0.01	0.91 ± 0.12	0.07 ± 0.02
	25 min	4.23 ± 1.06	0.06 ± 0.01	0.83 ± 0.10	0.07 ± 0.01
	30 min	3.63 ± 0.95	0.06 ± 0.02	0.80 ± 0.11	0.08 ± 0.02
Week 8					
	0 min	2.72 ± 0.55	0.06 ± 0.01	0.76 ± 0.13	0.06 ± 0.01
	3 min	5.94 ± 2.39	0.05 ± 0.01	0.79 ± 0.19	0.06 ± 0.01
	5 min	5.72 ± 3.25	0.06 ± 0.01	0.80 ± 0.13	0.08 ± 0.02
	25 min	4.09 ± 1.72	0.06 ± 0.01	0.79 ± 0.11	0.07 ± 0.01
	30 min	3.62 ± 1.44	0.09 ± 0.02	1.03 ± 0.22	0.06 ± 0.01

Data are presented as means ± SEs.

### 3.4 Muscle activity

There was a significant main effect of side (*p* < 0.0001) and side by week interaction (*p* = 0.0209) on ARV in the *masseter* (**Table 7**). ARVs were higher on the left than on the right side (*p* < 0.0001). ARVs were higher on the right side in week 1 than on the left side in weeks 4, 5, and 8 (*p* ≤ 0.0421). ARV on the left side in week 1 was higher than on the right side (*p* = 0.0023). There were no significant differences in MF of the *masseter* (**Table 8**).

**Table 7:** Average rectified values (ARV) from the *masseter*, *sternocleidomastoid*, and *cervical trapezius* muscles.

	*Masseter*		*Sternocleidomastoid*		*Cervical Trapezius*	
Week	Left	Right	Left	Right	Left	Right
1	13 ± 3^a^	9 ± 3^b^	32 ± 14^b,y,z^	15 ± 5^a,y^	24 ± 5	23 ± 7
2	7 ± 1	6 ± 2	13 ± 2^y,z^	12 ± 2^y,z^	15 ± 3	14 ± 3
3	6 ± 1	6 ± 1	25 ± 4^y^	19 ± 4^z^	68 ± 31	89 ± 33
4	9 ± 2	5 ± 1	20 ± 2^y,z^	26 ± 5^z^	48 ± 16	37 ± 12
5	9 ± 2	6 ± 1	46 ± 30^z^	25 ± 15^y,z^	20 ± 5	19 ± 3
6	7 ± 2	7 ± 2	33 ± 11^y^	14 ± 3^y,z^	27 ± 11	13 ± 3
7	12 ± 6	8 ± 2	29 ± 13^y,z^	11 ± 2^y,z^	21 ± 9	57 ± 18
8	7 ± 1	7 ± 2	35 ± 11^y^	37 ± 12^z^	6 ± 1	8 ± 2

Data are presented as means ± SEs. ^a,b^Values within a row with differing superscripts are significantly different (*p* < 0.05). ^y,z^Values within a column with differing superscripts are significantly different (*p* ≤ 0.05).

**Table 8:** Median frequency (MF) from the *masseter*, *sternocleidomastoid*, and *cervical trapezius* muscles.

	*Masseter*		*Sternocleidomastoid*		*Cervical Trapezius*	
Week	Left	Right	Left	Right	Left	Right
1	8 ± 1	8 ± 1	7 ± 1	7 ± 1	8 ± 1	9 ± 1
2	7 ± 1	9 ± 1	10 ± 1	9 ± 1	7 ± 1	6 ± 1
3	8 ± 1	11 ± 2	8 ± 1	8 ± 1	8 ± 1	9 ± 1
4	9 ± 1	8 ± 1	8 ± 1	10 ± 2	9 ± 1	9 ± 1
5	10 ± 2	8 ± 1	10 ± 1	9 ± 1	8 ± 1	8 ± 1
6	8 ± 1	8 ± 1	7 ± 1	8 ± 1	8 ± 1	9 ± 1
7	7 ± 1	11 ± 2	8 ± 1	8 ± 1	8 ± 1	9 ± 1
8	9 ± 1	10 ± 1	8 ± 1	8 ± 1	8 ± 1	8 ± 1

Data are presented as means ± SEs.

There were significant main effects of week (*p* < 0.0001) and side (*p* = 0.0025) and side by week interaction (*p* = 0.0071) on ARV in the *sternocleidomastoid* (**Table 7**). ARV in week 1 was lower than weeks 3, 4, and 8 (*p* ≤ 0.0067). ARV in week 5 was lower than weeks 3, 4, and 8 (*p* ≤ 0.0236). ARV in week 7 was lower than weeks 3 and 8 (*p* = 0.0130 and 0.007). ARV was lower on the right side than on the left (*p* = 0.0025). In week 1, ARVs were higher on the left side than on the right (*p* = 0.0111). ARV on the left side in week 5 was higher in weeks 3, 6, and 8 on the left side and week 8 on the right side (*p* ≤ 0.0436). ARVs were higher on the left side in week 3 than the right side in weeks 1, 6 and 7 (*p* ≤ 0.0152). ARV on the right in week 1 was lower than on the left side in weeks 4, 6, and 8 and on the right side in weeks 3, 4, and 8 (*p* ≤ 0.0034). ARV on the left side in week 8 was higher than on the right side in weeks 6 and 7 (*p* = 0.0454 and 0.0449). There were no statistically significant differences in MF of the *sternocleidomastoid* (**Table 8**).

There was a significant main effect of week (*p* < 0.0001) on ARV in the *cervical trapezius* (**Table 7**). ARVs in week 8 were lower than in weeks 1, 3, 4, and 5 (*p* ≤ 0.0188). ARV in week 3 were higher than in weeks 2 and 6 (*p* = 0.0089 and 0.0008). ARVs in week 5 were higher than week 6 (*p* = 0.0170). There were no statistically significant effects on MF in the *cervical trapezius* (**Table 8**).

### 3.5 Human–horse interaction

**Figure 4:**
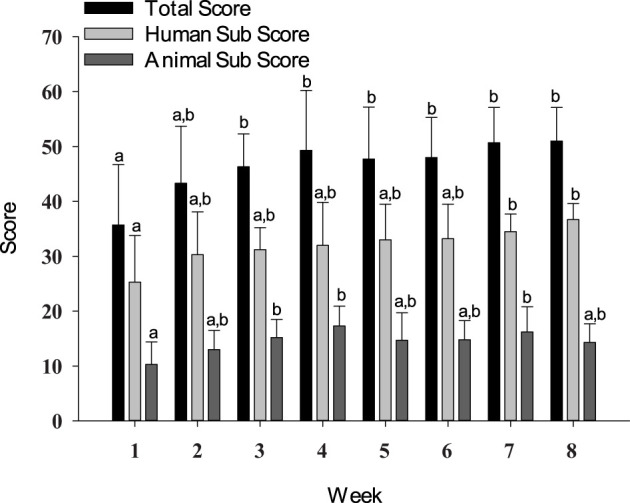
Total score, human subscore, and animal subscore from the Human–Animal Interaction Scale. Data are presented as means ± SEs. Differing superscripts indicate statistically significant differences.

Human subscores on the Human–Animal Interaction Scale were higher in weeks 7 (*p* = 0.0151) and 8 (*p* = 0.0013) as compared to that in week 1. Animal subscores were higher in weeks 3 (*p* = 0.0362), 4 (*p* = 0.0006), and 7 (*p* = 0.0059) as compared to that in week 1. Total scores were higher (*p* ≤ 0.0437) starting in week 3 and remained higher through week 8 as compared to week 1 scores (**Figure 4**).

## 4 Discussion

The objectives of the study were to 1) determine the feasibility of recruiting, enrolling, and completing data collection with veterans with PTSD; 2) explore the effects of adaptive horsemanship lessons on symptoms of PTSD, hormone concentrations, and social motor synchrony in combat veterans; 3) determine if physiological changes occur as veterans interact with horses in weekly lessons; and 4) explore if the interaction between veteran and horse changes over the course of an 8-week session. Only nine veterans completed data collection, indicating that the recruitment efforts and enrollment process should be modified. All data collection procedures were implemented without adverse effects, and the preliminary results support further investigation into equine-assisted services for veterans with PTSD.

### 4.1 Participant attrition

High rates of potential participant attrition occurred in this study with 84% of potential participants being lost or excluded from the study between initial contact and completion of informed consent. While higher than the attrition rate reported by Fisher et al. (12) in their study of equine-assisted psychotherapy for veterans with PTSD (66%), it would seem that a high rate of attrition is to be expected in these types of studies, particularly if attrition rate is calculated from initial contact. In the present study, this high attrition rate contributes to the low number of participants completing data collection, which is a severe limitation and makes this a pilot study. High attrition rates and low therapy session attendance are a common problem plaguing research involving human participants (5, 56). Attrition rates in the current study were further complicated by the need for pair-matched control participants. Researchers planning to work with human participants, particularly this population of veterans with PTSD, need to account for these high attrition rates in their study design and recruitment efforts. While some participants were lost after completing the informed consent process, no participants were lost after starting the AH intervention. This is a promising finding given the high rates of dropout associated with traditional therapies for veterans with PTSD and has been corroborated by other researchers (5, 13).

The high attrition rate observed in the beginning stages of the enrollment process may have been compounded by using the CAPS-5 to determine PTSD diagnosis and eligibility for enrollment in the study. Anecdotally, some potential participants had previously received a PTSD diagnosis but failed to meet the criteria for a past-month diagnosis of PTSD using the CAPS-5 at the time of potential enrollment in the study. This discrepancy could be due to a multitude of factors including a diminishment of symptoms since the previous diagnosis, differences in diagnostic criteria, and underreporting of symptoms during the enrollment process. In the future, researchers may want to consider using the lifetime CAPS-5 instead of the past month CAPS-5 or accepting external proof of past PTSD diagnosis to help combat high attrition. Low enrollment in this study was the primary challenge in conducting the study.

### 4.2 PTSD symptoms

The decrease in depression, hostility, and paranoid ideation as measured on the BSI and the tendency for cognition and mood altering symptoms as measured on the PCL-5, and somatization, and psychoticism as measured on the BSI, and overall scores on both instruments to decrease are in line with outcomes reported in other studies, which also reported decreases in PTSD symptoms following various forms of equine-assisted services (8–13, 21). Variation (SE) in scores for PTSD symptoms in the current study was numerically higher than that reported by Malinowski et al. (8) and closer to that reported in the other studies. This is likely due to the time frame of the studies as the study of Malinowski et al. (8) occurred over 5 days and the other studies occurred over periods of weeks as did this study. With the exception of Malinowski et al. (8), these studies only reported overall scores on the PCL-5 or PTSD Checklist—Military Version (PCL-M), although all reported significant decreases in PCL-5 or PCL-M scores. Malinowski et al. (8) reported significant decreases in overall scores on both the PCL-5 and BSI. Hyperarousal symptoms were the only category of symptoms on the PCL-5, which showed significant reductions, while all symptom subcategories except for phobic anxiety and interpersonal sensitivity on the BSI showed significant decreases in the study of Malinowski et al. (8). The disparity in symptom clusters or subcategories showing change across studies is worth investigating further. Different types of equine-assisted services may be more or less effective in addressing particular clusters of symptoms, as the two studies in question employed different approaches—adaptive horsemanship lessons vs. psychotherapy integrating horses. While the changes seen in the current study are insufficient to guide treatment decisions, they do support continued investigation of equine-assisted services as a treatment for PTSD.

Studies by Arnon et al. (13), Fisher et al. (12), Lanning et al. (22, 26), and Marchand et al. (24) have reported data from follow-up times points ranging from 30 days to 3 months after the intervention. Except for Arnon et al. (13), these studies reported persistence of symptom reduction at the follow-up time point. In the current study, obsessive–compulsive, anxiety, and paranoid ideation subscores on the BSI were lower at the 2-month follow-up than at the pre-intervention time point. The preliminary results obtained in the current study and results from other studies reporting data from follow-up time points lead us to suggest that future studies should include measurements at follow-up time points to more closely track how symptoms change during the period following cessation of the activity or therapy. This is valuable information for providers and clinicians to guide their decisions about intervention duration and cessation.

### 4.3 Social motor synchrony

A circular variance value of 1 represents perfect synchrony, and a value of 0 represents no synchrony. Overall, circular variance was lower than values reported in other studies (37, 38). The reason for this discrepancy is unclear and may have contributed to the lack of differences observed at pre- and post-measurement time points. It should be noted that similarly low values were also obtained from a group of veterans without PTSD (57). While researchers may wish to explore this measure further in future studies, the difficulty of locating pair-matched individuals for the dyadic task limits the feasibility of this measure.

### 4.4 Hormone concentrations

All values for cortisol, epinephrine, and norepinephrine concentrations during all measurement time points fell within or only slightly above normal reference ranges for these hormones (58). Plasma oxytocin concentrations were similar to those reported by authors using the same quantification methods; however, there is debate over the accuracy of these quantification methods (59). Dilution recommendations provided by the manufacturer of the ELISA kit were followed. The lack of change in basal plasma hormone concentrations (cortisol, epinephrine, norepinephrine, and oxytocin) is likely related to the fact that these hormone concentrations were within normal ranges at the beginning of the study and did not differ significantly from the healthy participants and could be confounded by the small sample size (57).

Oxytocin, cortisol, and norepinephrine plasma concentrations remained relatively stable across session timepoints and weeks in the veterans. As this is the first study to have measured these hormones in this population during sessions, comparison among studies is impossible. The social bonding at play during humans’ interactions with dogs that leads to increases in oxytocin concentrations may not be present in human–horse interactions, although further work is needed to confirm this hypothesis (60, 61). The response of the hypothalamic–pituitary–adrenal (HPA) axis also appeared to be unaffected by veterans’ interactions with horses, as no changes in cortisol concentrations were found. Responses of the autonomic nervous system are not as straightforward, as no changes in norepinephrine concentrations were found, but epinephrine tended to decrease between weeks 1 and 4. High concentrations of epinephrine in week 1 and the subsequent decrease lead us to suggest that veterans experienced a shift toward sympathetic activation in the autonomic nervous system during week 1 and that in later weeks, the balance shifted toward parasympathetic activation. Given that anxiety and heightened arousal, especially in unfamiliar situations, is a component of PTSD, it would seem reasonable to expect veterans to experience these types of responses in entering an unfamiliar environment with novel expectations (62, 63). Given the small sample size, these are preliminary results and should only be used in guiding the development of future research studies.

Previous research has failed to find links between salivary and plasma catecholamines, making salivary measurement of these hormones scientifically unsound (64, 65). Thus, if researchers wish to continue pursuing this line of investigation, blood sampling will be needed. The sampling procedures (catheter) used in this study resulted in no adverse events, and no participants voiced concern over the procedures. The samples were quickly drawn with the veteran seated during the procedure. While further spacing between sample time points would be recommended; in this population, the procedure was feasible to implement. The fact that all participants were adults and a small sample was taken at each time point contributed to the feasibility of the blood draws. Additionally, a registered nurse with extensive experience in conducting blood draws for research studies was employed for these procedures and contributed to the success of the blood draws.

### 4.5 Muscle activity

Changes in ARV in the *sternocleidomastoid* and *cervical trapezius* muscles are likely an artifact of the veterans’ movements during the lessons, as EMG traces for these muscles were visually observed to change with the veteran’s movement and activity during live monitoring of data collection. These same patterns were not visually apparent in the EMG trace of the *masseter.* Results should still be interpreted with caution as recordings occurred during anisometric, dynamic movements rather than the isometric, static movements usually used for robust interpretation of results (66). ARV values in the current study were lower than those generally reported in the literature (67, 68). It should be borne in mind, however, that most research using EMG measures report results from targeted, specific activities or movements, whereas the current study included data from periods of relatively low activity when low muscle activation would be expected. Measuring sEMG as a potential marker of stress via muscle tension in an applied context seems unlikely to be useful given the results of this study.

### 4.6 Human–horse interaction

This study is the first known report of using the Human–Animal Interaction Scale with horses, as it was developed with other animal species (40). The increases in overall scores starting in week 3 were expected as Wharton et al. (11) also reported increases in client–therapist and client–horse relationships following psychotherapy incorporating horses. The Human–Animal Bond Scale used by Wharton et al. (11) had not been validated, however. The increase in scores in the present study indicates more positive human–horse interactions, as higher scores on the Human–Animal Interaction Scale are indicative of more positive behaviors, fewer negative behaviors, or both (40). The changes in horse subscores and total scores did not appear to be related to measurable behavioral changes in the horses, as no differences in horse behavior were found (41). The behavioral measures used in this study did not account for affiliative behaviors, though, which would be included in the Human–Animal Interaction Scale. Future studies should look at behavior and scores on instruments like the Human–Animal Interaction Scale, as there may also be differences in self-report and researcher reported measures as perception can become a confounding variable. In making decisions about which instrument to use, researchers should carefully consider whether the scale incorporates human behavior, animal behavior, or both. It may be useful to develop a scale specific to horse–human interactions, as behavior does vary across species.

### 4.7 Conclusion

The preliminary results of this pilot study support the need for further research addressing the outcomes of equine-assisted services for veterans with PTSD. The results from this small pilot study contribute to the literature that reports positive outcomes in terms of symptom severity following equine-assisted services. It also demonstrates the feasibility of collecting physiological data, such as hormone concentrations, during lessons without adverse effects for the participants. Recruitment was the greatest challenge in this study, and researchers planning to pursue this line of research should carefully plan to mitigate this challenge.

## Data availability statement

The datasets presented in this article are not readily available because: Participants did not provide consent for sharing of data during the informed consent process. Requests to access the datasets should be directed to ellen.rankins@colostate.edu.

## Ethics statement

The studies involving humans were approved by Rutgers University Institutional Review Board. The studies were conducted in accordance with the local legislation and institutional requirements. The participants provided their written informed consent to participate in this study. The animal studies were approved by Rutgers University Institutional Animal Care and Use Committee. The studies were conducted in accordance with the local legislation and institutional requirements. Written informed consent was obtained from the owners for the participation of their animals in this study.

## Author contributions

ER: Conceptualization, Data curation, Formal analysis, Funding acquisition, Investigation, Methodology, Project administration, Visualization, Writing – original draft. AQ: Methodology, Resources, Supervision, Writing – review & editing. KHM: Conceptualization, Methodology, Supervision, Writing – review & editing. KM: Conceptualization, Funding acquisition, Methodology, Supervision, Writing – review & editing.
